# Wellbeing in and Through Performance: Perspectives From Sports and Music

**DOI:** 10.3389/fpsyg.2020.00399

**Published:** 2020-03-06

**Authors:** Aaron Williamon, Roberta Antonini Philippe

**Affiliations:** ^1^Centre for Performance Science, Royal College of Music, London, United Kingdom; ^2^Faculty of Medicine, Imperial College London, London, United Kingdom; ^3^Laboratoire PHASE, Faculté des Sciences Sociales et Politiques, Institut des Sciences du Sport, Université de Lausanne, Lausanne, Switzerland

**Keywords:** mental skills, music, performance preparation, sports, wellbeing

Each day, millions of people perform. Whether in the boardroom, classroom, courtroom, laboratory, operating theater, on the field or on stage, performances are almost invariably dynamic and, in some cases, extremely rewarding. Nonetheless, each performance also brings challenges, real and perceived, that may lead to patterns of negative thinking, avoidance behavior, and debilitating injury that can have serious consequences for success and for health.

In this article, we draw insight and inspiration from sports to focus on how elite performers from other fields—and in particular, music—can come to integrate healthy approaches to performing alongside their drive to succeed.

## Linking Performance and Wellbeing: Directions in Sports

The popularity of sports psychology has grown substantially in the past two decades, and the importance of being mentally prepared prior to an athletic competition is now well-documented (von Treuer and Reynolds, 2017). Historically, sports psychology came about through the specific intention to *enhance* performance, but over time, the need to focus on athlete wellbeing as an integral part of performance has come sharply into focus (Sebbens et al., 2016). Indeed, very early in the field's development, negative consequences of performing at the highest levels–injuries, eating disorders, performance anxiety, occupational stress, and so on— surfaced in ways that were shown to influence directly how athletes lived and performed (MacIntyre et al., 2017). In other words, athletes face multiple and varied challenges that are inherent to their training and their work. When studying these phenomena, the self-regulation efforts that individuals use to alter their interaction with the environment to better meet their goals also need to be considered (Lazarus and Folkman, 1984). To be well prepared for a performance, emotions too should be self-regulated during the preparation and learning process. Foundational research into coping skills for managing competitive situations has emerged (Gould et al., 1993), with *coping* defined as the process of using “cognitive and behavioral effort to manage specific external and/or internal demands that are appraised as taxing or exceeding the resources of the person” (Lazarus and Folkman, 1984, p. 141). Research has demonstrated that failure to cope with acute stressors can have a detrimental impact on psychological processes and psychological balance (Smith, 1986). Positive coping strategies, conversely, offer wide-ranging benefits that facilitate performance success, which can be developed and reinforced through mental (or psychological) skills training.

While there is a tendency to think of certain psychological strengths as necessary only in moments of crisis, mental skills are, in fact, cited as one of the *essential* components of athletic performance across the board (Durand-Bush and Salmela, 2002). While it is true that mental skills can help manage disruptive factors that impede or impair optimal performance, training to develop mental skills is central to personal development and to the enhancement of both performance and wellbeing throughout an athlete's career (Ducasse and Chamalidis, 2006).

## Lessons Learned From Sports: Perspectives on Advancing Performers' Health and Wellbeing in Music

Wellbeing is a multidimensional phenomenon and refers to emotional and cognitive dimensions of subjective experiences resulting from the individual evaluation of several facets of life (Disabato et al., 2016). Considering the link between performance and wellbeing established in sports, some researchers have focused on implications for other performance domains. A particular emphasis has been placed on the arts, and music specifically, for the parallels between sport and music performance across a wide array of psychosocial demands that performers in both fields must manage (Williamon, 2004; Antonini Philippe and Güsewell, 2016).

The studies highlight musicians' use, largely, of insufficient adaptation strategies to achieve positive health (Antonini Philippe, 2013; Antonini Philippe and Güsewell, 2016). A growing body of research shows that specific stressors and demands that musicians face in their training can manifest in performance-related pain and discomfort, performance anxiety, and occupational stress, all of which can be detrimental to wellbeing and pose significant barriers to performing effectively (Williamon and Thompson, 2006; Cruder et al., 2018).

Recently, studies have focused on a more *positive* psychological approach to performance in order to encourage and reinforce health-promoting behaviors in conservatoires and schools of music (see Ascenso et al., 2017, 2018; Perkins et al., 2017). One study (Antonini Philippe et al., 2019) revealed that, for those students who commit to music professionally, more action is needed to support their health directly and to bolster the value placed on health, both by the musicians themselves as well as their teachers, administrators, and support staff. The results also highlight exciting new possibilities for intervention programs aimed at assisting musicians in drawing closer ties between improving health and enhancing performance, many of which have already been piloted and applied in sporting contexts (see Williamon et al., 2017).

Further studies are now underway, for instance examining pre-performance routines and effective methods for recovering from acute stress, with the principal aim of developing musician-tailored mental skills training programs. By this, we mean “the systematic and consistent practice of mental or psychological skills for the purpose of enhancing performance, increasing enjoyment, or achieving greater… self-satisfaction” in performance (Weinberg and Gould, 2007, p. 250). We believe that progress in this area is as essential for musicians now as it has been for athletes over the past two decades. In doing so, we must be open to the prospects of knowledge transfer from the arts back to sports too, and onwards to other performance specialisms.

## Next Steps

We recognize that there is a complex interaction between health and performance, and we suggest that future research must interrogate attitudes, behaviors, and indicators of well- and ill-being, with the aim of fostering positive approaches to training and performance both within single disciplines and, where appropriate, across them.

In music, much is now happening to facilitate dialogue and apply research outcomes in training contexts (see Ginsborg et al., 2009; Wasley et al., 2012; Clark et al., 2013; Perkins et al., 2017). For instance, the *Musical Impact* project (funded by the United Kingdom's Arts and Humanities Research Council) revealed a strong desire—from musicians themselves, as well as those who train and employ them—for close collaboration in supporting and enhancing the health of performing artists (Araújo et al., 2017, 2020). The research team consequently worked with partners across the arts to constitute *Healthy Conservatoires*, an international network bringing together key stakeholders to share information, research, and good practice for advocating and advising on health in educational and professional settings^1^.

Beyond the arts, performance scientists must seek to shape wider public health agendas, particularly as their research relates to the challenges and demands that people face each day when they perform. In this way, we will be well placed to identify ways in which performers in all sectors of society can thrive in their chosen activities, over sustained careers.

## Author Contributions

All authors listed have made a substantial, direct and intellectual contribution to the work, and approved it for publication.

### Conflict of Interest

The authors declare that the research was conducted in the absence of any commercial or financial relationships that could be construed as a potential conflict of interest.

## Footnotes

^1^www.HealthyConservatoires.org
